# *EGFR* amplification is a putative resistance mechanism for NSCLC–LM patients with TKI therapy and is associated with poor outcome

**DOI:** 10.3389/fonc.2022.902664

**Published:** 2022-08-01

**Authors:** Hainan Yang, Lei Wen, Chao Zhao, Xuefei Li, Changguo Shan, Da Liu, Weiping Hong, Zhaoming Zhou, Cheng Zhou, Linbo Cai, Caicun Zhou

**Affiliations:** ^1^ Department of Oncology, Shanghai Pulmonary Hospital and Thoracic Cancer Institute, School of Medicine, Tongji University, Shanghai, China; ^2^ Department of Oncology, Guangdong Sanjiu Brain Hospital, Guangzhou, China; ^3^ Department of Neurosurgery, Guangdong Sanjiu Brain Hospital, Guangzhou, China

**Keywords:** non-small cell lung cancer, cerebrospinal fluid, leptomeningeal metastases, *EGFR* gene amplification, EGFR-TKI

## Abstract

**Background:**

Leptomeningeal metastases (LM) have become increasingly common in non-small cell lung cancer (NSCLC) patients who harbor epidermal growth factor receptor (*EGFR*) mutation treated with *EGFR*-TKI and are correlated with inferior prognosis. Evidence in prior research demonstrated that *EGFR* amplification was more likely presented in advanced clinical stages and was associated with worse survival. However, whether *EGFR* amplification is a prognostic marker in NSCLC–LM is still inconclusive.

**Methods:**

This study enrolled patients diagnosed with NSCLC–LM from June 2019 to September 2021 and who had received previous EGFR-TKI at Guangdong Sanjiu Brain Hospital. Cerebrospinal fluid (CSF) samples were collected and subjected to targeted next-generation sequencing of 168 cancer-related genes. Clinical characteristics and overall survival (OS) were compared in patients with and without *EGFR* amplification.

**Results:**

This study enrolled 53 NSCLC–LM patients, all of whom had *EGFR* mutations. *TP53* and *EGFR* amplifications are the two most frequent mutations in the study cohort, presenting at 72% (38 of 53) and 40% (21 of 53), respectively. The rate of *EGFR* amplification was much higher at the time of leptomeningeal progression than at initial diagnosis (*p* < 0.01). Karnoskfy performance status was poorer (*p* = 0.021), and CSF pressure was higher (*p* = 0.0067) in patients with *EGFR* amplification than those without. A multivariable Cox proportional hazard regression model showed that *EGFR* amplification was an independent prognostic factor for poorer OS (8.3 *vs*. 15 months; *p* = 0.017). The median OS was shorter in NSCLC–LM patients with mutated *TP53* than those with wild-type *TP53*, but the difference was not statistically significant (10 *vs*. 17.3 months, *p* = 0.184).

**Conclusions:**

*EGFR* gene amplification could be a potential resistance mechanism to *EGFR*-TKI failure in NSCLC–LM and is associated with inferior clinical outcomes.

## Introduction

Lung cancer is one of the most frequently diagnosed types of cancer (1). Although the 5-year overall survival (OS) rate for patients at all stages of lung cancer is 19%, most patients are diagnosed with advanced-stage disease, with the latter patients having a 5-year OS rate of only 3% (2, 3). The development of epidermal growth factor receptor (*EGFR*) tyrosine kinase inhibitors (TKIs) has significantly prolonged the survival of selected patients who harbor *EGFR* mutations when compared with platinum-based combination chemotherapy (4, 5). However, the efficacy of EGFR-TKIs is likely limited by innate or acquired resistance. Osimertinib is a third-generation EGFR-TKI designed to overcome the resistance to other TKIs of tumors harboring *EGFR*-T790M (6, 7). The median progression-free survival (PFS) was 9.6 months for patients who were *EGFR* T790-positive and who had a progressive disease after a previous TKI treatment (8).

Leptomeningeal metastases (LM) of lung cancer have been associated with poor prognosis. LM has been observed in 9.4% of patients with *EGFR* mutation, compared with 1.7% of patients without *EGFR* mutations (9). Osimertinib has been shown to penetrate the blood–brain barrier in animal models (10) and has shown promising therapeutic efficacy in NSCLC–LM patients resistant to prior EGFR-TKI therapy (11). Resistance to osimertinib develops over time, although the potential resistance mechanism remains unclear. *EGFR* protein overexpression was much higher in metastatic lesions than in primary tumors, with high gene copy numbers indicating tumor progression (12). The association between *EGFR* amplification and prognosis in patients with NSCLC–LM has not been determined.

## Patients and methods

### Patients

The present study included 53 patients diagnosed with NSCLC–LM who were enrolled between June 2019 and September 2021 at Guangdong Sanjiu Brain Hospital. LM was diagnosed based on enhanced MRI results showing a linear or micronodular pial enhancement, as assessed by two experienced radiologists, or the detection of tumor cells in cerebrospinal fluid (CSF) samples. The patients were divided into groups, one with and the other without *EGFR* amplification.

### Methods

Approximately 10 ml of CSF was obtained from each patient through a lumbar puncture at the time of leptomeningeal progression. Targeted next-generation sequencing (NGS) was performed to detect somatic mutations in a panel of 168 cancer-related genes. Genomic profiles were assessed using the core panel from Burning Rock Biotech (Guangzhou, China). Cytology findings, opening pressure of CSF, and Karnoskfy performance status (KPS) score were evaluated by the treating physician. The protocol of this study was approved by the Research Ethics Committee of Guangdong Three Nine Brain Hospital.

### Statistical analysis

Continuous variables were reported as arithmetic medians or means and categorical variables as proportions with 95% confidence intervals (CIs). Differences between patients with and without *EGFR* amplification were evaluated by Pearson chi-square test, Fisher’s exact test, or Wilcoxon test. OS, defined as the time period from the date of diagnosis of LM to the date of death or last follow-up, was assessed by the Kaplan–Meier method and compared by log-rank tests. Statistical analyses were performed using SPSS 21, Graph Pad Prism 6, and R version 4.1.2 software, with *p <*0.05 defined as statistically significant.

## Results

### Clinicopathologic features of patients with NSCLC–LM

A total of 53 NSCLC patients with leptomeningeal progression were enrolled in this study, including 21 (40%) with and 32 (60%) without *EGFR* gene amplification. The demographic and clinical characteristics, mutation profiles, and treatment history of these patients are summarized in **Table 1**. The median age of the 53 patients was 56 years (range, 36 to 74 years) and was similar in the patients with (median age, 51 years; range, 38 to 72 years) and without (median age, 56.5 years; range, 36 to 74 years) *EGFR* amplification. The 53 patients included 24 (45%) women, with similar percentages in patients with (43%; 9/21) and without (47%; 15/32) *EGFR* amplification. The median KPS was 70 (range, 30–90) and was significantly lower in patients with than those without *EGFR* amplification (*p* = 0.021).

**Table 1:** Clinical characteristics of 53 non-small cell lung cancer–leptomeningeal metastases (LM) patients with EGFR mutation.

Characteristics	Study cohort Number (%)	With EGFR-amp(cohort 1)Number (%)	Without EGFR-amp(cohort 2)Number (%)
Number of patients	53	21	32
Age (years), median (range)	56 (36–74)	51 (38–72)	56.5 (36–74)
Gender			
Male	29 (55)	12 (57)	17 (53)
Female	24 (45)	9 (43)	15 (47)
Karnoskfy performance status, median (range)	70 (30–90)	60 (30–90)	75 (40–90)
*EGFR* mutation status			
19del	20 (38)	8 (38)	12 (38)
L858R	29 (55)	10 (47)	19 (59)
20ins	2 (4)	2 (10)	
L861Q	2 (4)	1 (5)	1 (3)
Other	2 (4)	1^a^	1^b^
T790M	5 (9)	1 (5)	4 (13)
Diagnosis of LM			
Positive cerebrospinal fluid (CSF) cytology	35 (66)	14 (67)	21 (66)
Typical brain imaging	18 (34)	7 (33)	11 (34)
Previous EGFR-TKI			
TKI—1st or 2nd	15 (28)	5 (24)	10 (31)
Gefitinib	8 (15)	2 (9)	6 (19)
**Icotinib**	4 (8)	1 (5)	3 (9)
**Erlotinib**	2 (4)	1 (5)	1 (3)
Afatinib	1 (2)	1 (5)	
TKI—3^rd^	38 (72)	16 (76)	22 (69)
Osimertinib	36 (68)	15 (71)	21 (66)
Almonertinib	2 (4)	1 (5)	1 (3)
CSF pressure (mmH_2_O)	172.5 (60–330)	210 (60–330)	150 (65–300)

a A patient has a co-mutation of EGFR L858R and 25 missense_variant.

b A patient has co-existing EGFR L858R and 15 missense_variant.

EGFR, epidermal growth factor receptor; TKI, tyrosine kinase inhibitor.

A total of 55 *EGFR* mutations were detected in the 53 patients. Twenty-nine patients, 10 with and 19 without *EGFR* amplification, had L858R mutations in exon 21; 20 (8 and 12, respectively) had deletions in exon 19, two (one in each group) had L861Q mutations in exon 21, and two, both with *EGFR* amplifications, had insertions in exon 20. Two patients had coexisting *EGFR* mutations: one, with an *EGFR* amplification, had the L858R mutation and a 25 missense_variant, and the other, without an *EGFR* amplification, had the L858R and a 15 missense_variant. Thirty-five (66%) patients had positive cytology in CSF samples, and all 53 had been treated with *EGFR* TKIs, including 38 (72%) who had received a third-generation TKI. The median CSF pressure was 172.5 mmH_2_O (range, 60–330 mmH_2_O) and was significantly higher in patients with (210 mmH_2_O; range, 60–330 mmH_2_O) than those without (150 mmH_2_O; range, 65–300 mmH_2_O) *EGFR* amplification (*p* = 0.0067).

### Gene profiling of CSF samples from NSCLC–LM patients

**Figure 1:**
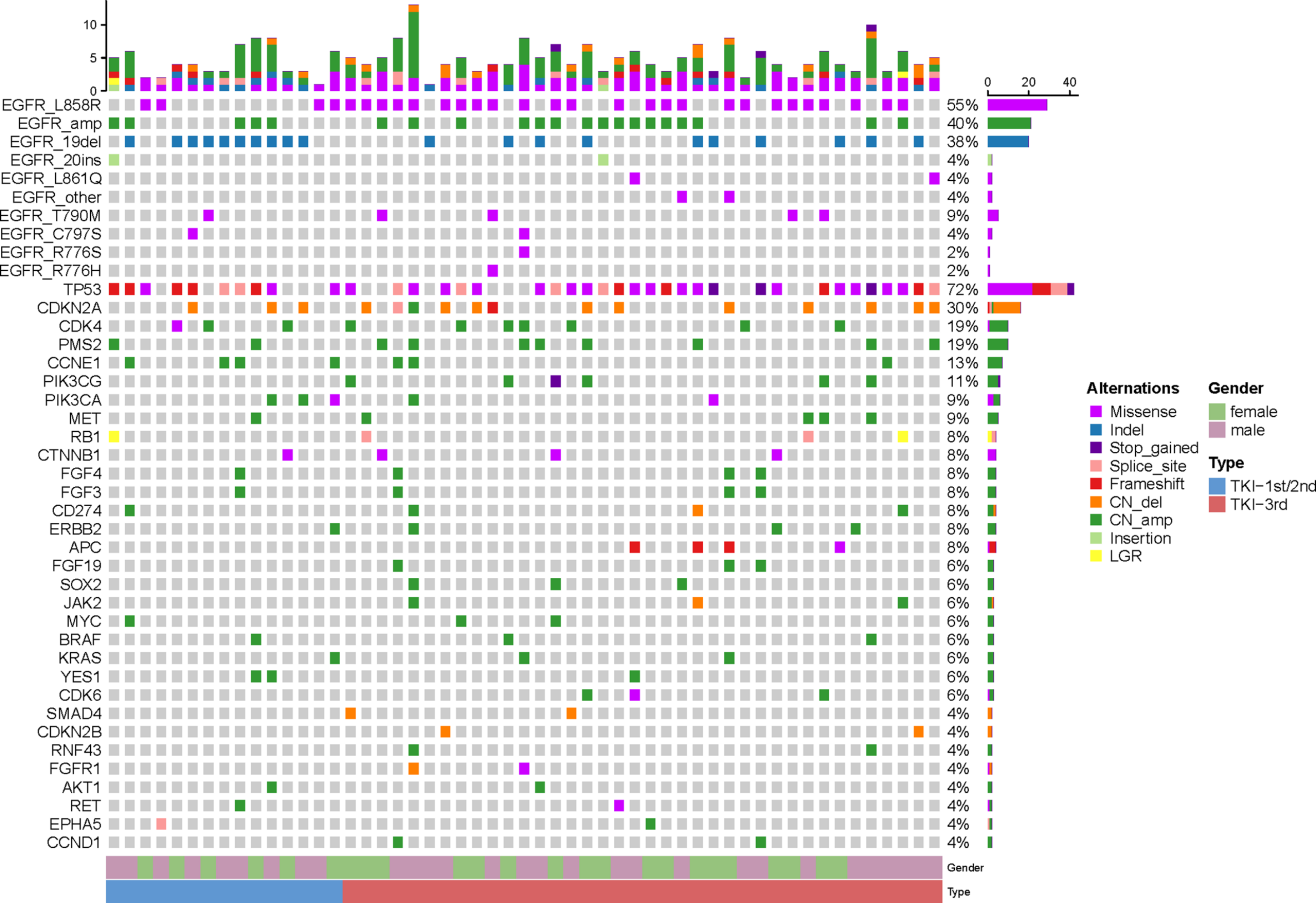
Next-generation sequencing results of 53 cerebrospinal fluid samples taken from non-small cell lung cancer patients with leptomeningeal metastases. The top bar shows the overall number of mutations in each patient. The right-side bar shows the percentage of patients harboring a specific mutation. Different colors denote different types of mutation. The bottom bar denotes patients grouped by gender or previous epidermal growth factor receptor tyrosine kinase inhibitor treatment history.

NGS at the time of LM showed *EGFR* mutations in 100% of the 53 CSF ctDNA samples, including L858R mutations in 29 (55%), exon 19 deletions in 20 (38%), exon 20 insertions in two (4%), and L861Q mutations in two (4%). *TP53* mutations and *EGFR* amplification were the two most frequent alterations in the study cohort, being present in 38 (72%) and 21 (40%) patients, respectively. In addition, *CDKN2A*, *PMS2*, and *CCNE1* mutations were detected in 16 (30%), 10 (19%), and seven (13%) patients, respectively. The rate of *EGFR* amplification was found to be higher in patients resistant to icotinib/gefitinib (13), with the present study finding that the rate of *EGFR* amplification was higher in patients treated with a third-generation TKI (42%, 16/38) than those treated with a first- or second-generation TKI (33%, 5/15). Moreover, patients with *EGFR* variants co-existing with *CDK4*, *CDK6*, and *MYC* mutations had poorer outcomes than those with *EGFR* variants alone (14). Of the 53 patients in this study, 10 (19%) had *CDK4* mutations in CSF samples, whereas three (6%) each had *CDK6* and *MYC* mutations (**Figure 1**).

An evaluation of mechanisms conferring resistance to *EGFR*-TKIs (**Table 2**) showed that the *EGFR* T790M and C797S mutations were present in five (9%) and two (4%) patients, respectively. In addition, five patients (9%) had *MET* mutations, four (8%) each had *RB1*, *ERBB2*, and *CTNNB1* mutations, and three (6%) had *KRAS* and *BRAF* mutations. The rates of detection of mutations in the *RB1*, *ERBB2*, *CTNNB1*, *BRAF*, and *KRAS* genes were similar in patients who have previously been treated with a third-generation TKI or a first- or second-generation TKI. In contrast, the detection rate of *CCNE1* mutations was about threefold higher in patients treated with a first- or second-generation than in those treated with a third-generation TKI (27 *vs*. 8%), indicating that *CCNE1* mutations may confer resistance to first- and second-generation TKIs. Conversely, the rates of detection of *EGFR* T790M and *MET* mutations were higher in patients treated with a third-generation TKI than those with a first- or second-generation TKI (11%, 4/38 *vs*. 7% 1/15 for both). In addition, mutations in *FGF19*, *CCND1*, and *SOX2* were detected only in patients treated with a third-generation TKI. Interestingly, NGS showed that seven patients have not less than three different mutation genes, and among them, three patients have not less than four mutation genes (**Table 2**).

**Table 2:** Potential resistance mechanism to EGFR-TKI in non-small cell lung cancer–leptomeningeal metastases.

Mutation type	Patients (*n* = 53)	1st/2nd (*n* = 15)	3rd (*n* = 38)
EGFR T790M	5 (9)	1 (7)	4 (11)
EGFR C797S	2 (4)	1 (7)	1 (3)
EGFR-amp	21 (40)	5 (33)	16 (42)
Alternative pathway activation			
Mutation of RB1	4 (8)	1 (7)	3 (8)
Mutation of MET	5 (9)	1 (7)	4 (11)
Mutation of ERBB2	4 (8)	1 (7)	3 (8)
Mutation of CTNNB1	4 (8)	1 (7)	3 (8)
Mutation of KRAS	3 (6)	1 (7)	2 (5)
Mutation of BRAF	3 (6)	1 (7)	2 (5)
Mutation of FGF3	4 (8)	1 (7)	3 (8)
Mutation of FGF4	4 (8)	1 (7)	3 (8)
Mutation of MYC	3 (6)	1 (7)	2 (5)
Mutation of CCNE1	7 (13)	4 (27)	3 (8)
Mutation of FGF19	3 (6)		3 (8)
Mutation of CCND1	2 (4)		2 (5)
Mutation of SOX2	3 (6)		3 (8)

Seven patients have not less than three different mutation genes in next-generation sequencing (NGS) testing. A patient has an increased copy number in five genes, such as FGF3, FGF4, FGF19, CCND1, and CCNE1. A patient has an increased copy number in four genes, including FGF3, FGF4, FGF19, and KRAS. Another patient also has an increased copy number in four genes, FGF3, FGF4, FGF19, and CCND1. Four patients have three mutation genes in the NGS results, including SOX2, CCNE1, and ERBB2 detected in a patient, SOX2, MYC, and CTNNB1 identified in a patient, KRAS, CCNE1, and ERBB2 found in a patient, and CCNE1, FGF3, and FGF4 shown in a patient.

### Comparison of characteristics in patients with and without *EGFR* amplification

**Figure 2:**
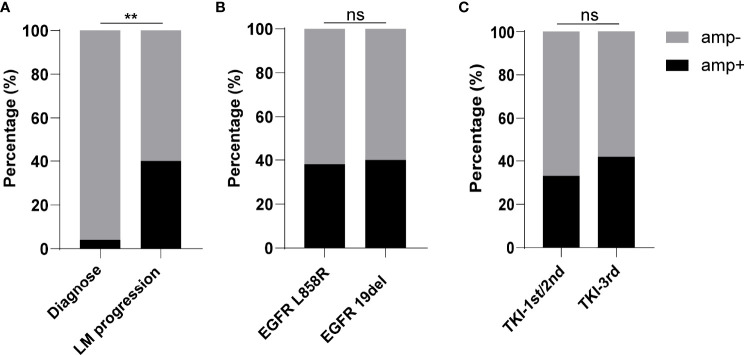
Characteristics of patients with or without epidermal growth factor receptor (EGFR) amplification. **(A)** Rate of detection of EGFR amplification at the time of initial diagnosis or leptomeningeal progression. **(B)** Rate of EGFR amplification in non-small cell lung cancer–leptomeningeal metastases patients harboring EGFR L858R or *EGFR* 19del. **(C)** Rate of *EGFR* amplification in patients previously treated with a first- or second-generation tyrosine kinase inhibitor (TKI) or a third-generation TKI. ** demonstrated p<0.01, and ns indicated no significance.

To identify the putative resistance mechanism to *EGFR*-TKI, we reasoned that gene aberrations should be absent from or have a low detection rate before treatment, with emergence or a high detection rate at progression. The initial gene profiles of 50 (94%) of the 53 patients showed that the detection rate of *EGFR* amplification was much higher at the time of leptomeningeal progression than at initial diagnosis (*p* < 0.01) (**Figure 2A**). This finding indicated that *EGFR* amplification could be associated with tumor progression in NSCLC patients with LM.

Deletions in exon 19 (19del) and mutations in exon 21 (L858R) are the two most common *EGFR* mutations. Although the rates of *EGFR* amplification were reported higher in lung adenocarcinoma patients with 19del than those with L858R mutations (15, 16), the present study found that *EGFR* amplification was present at similar percentages, being detected in 40% (8/20) of patients with 19del and 35% (10/29) of patients with L858R mutations (**Figure 2B**).

*EGFR* amplification is frequent in patients with acquired resistance to first- and second-generation TKIs (13), but less is known about the rate of amplification in patients resistant to third-generation TKIs. An evaluation of the clinical characteristics showed that the rate of *EGFR* amplification was slightly higher in patients treated with a third-generation than those with a first- or second-generation TKI, but the difference was not statistically significant (**Figure 2C**).

### *EGFR* amplification correlates with poorer prognosis

**Figure 3:**
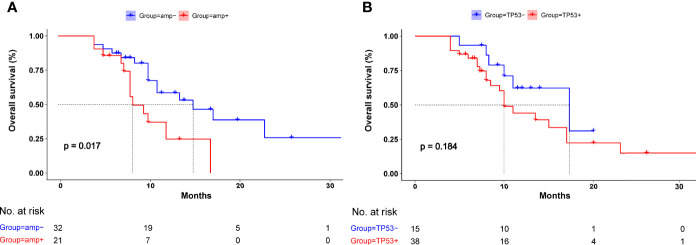
Kaplan–Meier analysis of overall survival in patients with and without **(A)** epidermal growth factor receptor (EGFR) amplification and **(B)** TP53 mutation. **(A)** Median overall survival was significantly poorer in patients with than those without EGFR amplification (*p* = 0.017). **(B)** Median overall survival was similar in patients with and without TP53 mutation.

**Figure 4:**
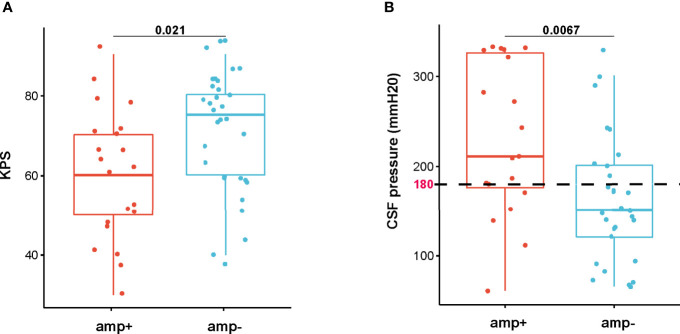
Distribution of **(A)** Karnoskfy performance status scores and **(B)** cerebrospinal fluid pressure in patients with and without epidermal growth factor receptor amplification.

The Kaplan–Meier analysis showed that the median OS was significantly shorter in patients with than those without *EGFR* amplification (8.3 *vs*. 15 months, *p* = 0.017, **Figure 3A**). The KPS scores were also significantly lower in patients with than those without *EGFR* amplification (*p* = 0.021, **Figure 4A**). The median CSF opening pressure was significantly higher in patients with than those without *EGFR* amplification (*p* = 0.0067, **Figure 4B**). In addition, open CSF pressure at or above the upper limit of the normal CSF range (80–180 mmH_2_O) was detected in 74% (14/19) of patients with but in only 31% (9/29) of patients without *EGFR* amplification. Moreover, Kaplan-Meier analysis showed that median OS was significantly shorter in patients with than without EGFR amplification (8.3 vs 15 months, p =0.017, **Figure 4A**).

An evaluation of the effect of *TP53* mutation and OS showed that the median OS was shorter in NSCLC–LM patients with mutated than those with wild-type *TP53*, although the difference was not statistically significant (10 *vs*. 17.3 months, *p* = 0.184, **Figure 3B**).

## Discussion

LM has become increasingly common in NSCLC patients harboring *EGFR* mutations and treated with *EGFR*-TKIs (17). The median OS in LM patients with *EGFR* mutations was found to be 8.9 months (95% CI: 7.2–10.7 months) (9). The potential mechanisms associated with poor prognosis remain unclear. *EGFR* amplification has been associated with significantly poorer outcomes in lung adenocarcinoma patients (15). Moreover, in addition to the *EGFR* T790M mutation, the rate of *EGFR* amplification was higher in patients with drug resistance than those with drug sensitivity (40 *vs*. 0%) (13). *EGFR* overexpression and high gene copy numbers have been associated with tumor progression in lung adenocarcinoma treated with *EGFR* inhibitors (12). Moreover, *EGFR* amplification is more likely to occur at advanced clinical stages and be associated with poorer disease-free survival (15). However, whether *EGFR* amplification is a prognostic marker in NSCLC–LM remains undetermined.

Previous research demonstrated that *TP53* was the most frequently mutated gene in CSF samples obtained from NSCLC patients with CNS metastases (14), and a similar study of NSCLC–LM patients who experienced disease progression on osimertinib also found that *TP53* was the most frequently detected concurrent gene in the CSF and that *EGFR* amplification and C797S mutation were also observed (18), shedding light on the potential resistance mechanisms among NSCLC–LM patients treated with *EGFR*-TKIs.

The present study compared 21 patients with *EGFR* mutations and concurrent *EGFR* amplification with 32 patients with *EGFR* mutations without concurrent *EGFR* amplification to assess the prognostic value of *EGFR* amplification in NSCLC–LM patients treated with *EGFR*-TKIs. OS was significantly shorter in patients with than those without *EGFR* amplification (*p* = 0.017). Moreover, patients with both *EGFR* and a co-mutation of amplification were more likely to have a poorer KPS score (*p* = 0.021) and a higher CSF pressure (*p* = 0.0067) than patients with a mutation but without *EGFR* amplification.

OS has also been reported as shorter in lung adenocarcinoma patients with than those without *TP53* mutations, and in patients with both *TP53* and *EGFR* mutations than in those lacking both (19). PFS was shown to be significantly longer in patients with mutated *EGFR* and wild-type TP53 than in patients with mutations in both genes (19 *vs*. 6.5 months, *p* = 0.035) 20). *TP53* was also the most frequently mutated co-occurring gene in our study cohort, being present in 72% of patients, with the median OS being shorter in NSCLC–LM patients with mutated than wild-type *TP53*, although the difference was not statistically significant (*p* = 0.184).

The *TP53* mutations were detected in 90% (19/21) of patients with and 59% (19/32) patients without *EGFR* amplification. To exclude the influence of *TP53* mutation on the result of this study, we performed a multivariate analysis to assess the prognostic significance of *EGFR* amplification. Univariable and multivariable Cox proportional hazard regression analyses were performed to assess the effects on survival of alterations in the *TP53*, *CDKN2A*, *CDK4*, *PMS2*, *CCNE1*, and *PIK3CG* genes, which all had a detection rate above 10% in the study cohort. The univariable analysis showed that *EGFR* amplification (*p* = 0.017) and *CDKN2A* mutation (*p* = 0.019) were significant predictors of OS, with multivariable analysis confirming that *EGFR* amplification [hazard ratio (HR), 2.63; 95% confidence interval (CI), 1.18–5.86; *p *= 0.018] and *CDKN2A* (HR, 2.61; 95% CI, 1.17–5.84; *p* = 0.019) were independent adverse predictors of OS. These findings indicate that *EGFR* amplification is an independent predictor of reduced OS, regardless of other genetic variations (**Table 3**).

**Table 3:** Univariable and multivariable analyses of gene signature detected in not less than 10% of the study cohort, and the results shown were obtained by performing the Cox regression model (*N* = 53).

Gene signature	Univariable analysis			Multivariate analysis		
	HR	95% CI	*p*-value	HR	95% CI	*p*-value
EGFR amp-	Reference					
EGFR amp+	2.29	1.0–5.25	**0.017**	2.63	1.18–5.86	**0.018**
TP53-	Reference					
TP53+	1.78	0.81–3.9	0.184			
CDKN2A-	Reference					
CDKN2A+	2.33	0.95–5.71	**0.019**	2.61	1.17–5.84	**0.019**
CDK4-	Reference					
CDK4+	1.02	0.41–2.53	0.962			
PMS2-	Reference					
PMS2+	0.67	0.27–1.71	0.440			
CCNE1-	Reference					
CCNE1+	2.13	0.59–7.63	0.105			
PIK3CG-	Reference					
PIK3CG+	0.51	0.17–1.52	0.330			

The bold values demonstrated the p<0.05 and were defined as statistically significant.

Additional coexisting mutations and the proportion of *EGFR* mutations can affect PFS (21), and immunohistochemical analyses of tumor tissue from NSCLC patients treated with first-line *EGFR*-TKIs have shown that co-occurring *PTEN* loss and *IGFR* overexpression correlated with poorer PFS and OS (22). In addition, the proapoptotic protein BIM and the negatively regulated apoptosis element of mTOR may account for the variable response of NSCLC patients to EGFR TKI therapy (23). These findings indicate that additional genetic alterations can affect the prognosis of patients treated with *EGFR* TKIs.

Evidence from prior research had shown that anti-*EGFR* antibody nimotuzumab could increase HLA class I expression in tumor cell lines (24), and another study found similar results showing that nimotuzumab can enhance NK cell activation and DC maturation and increase *EGFR*-specific T cell (25). A preclinical study found that, *in vitro*, nimotuzumab can enhance the radiotherapy effect in human esophageal squamous cell carcinoma cells, and this finding was also confirmed in cell xenografts, showing that radiation combined with nimotuzumab was correlated with tumor growth delay in contrast to radiation alone (26). Preclinical data have suggested that nimotuzumab enhances the antitumor activity, and evidence from several clinical trials further confirmed these findings. A phase 3 clinical trial in locally advanced head and neck cancer indicated that the addition of nimotuzumab could improve PFS when compared to the same schedule with weekly cisplatin (27). A similar research has also shown that adding another *EGFR* monoclonal antibody cetuximab indicated increased survival in advanced non-small-cell lung cancer patients compared with chemotherapy alone (28). Given the results showing a synergistic effect of nimotuzumab and chemotherapy or radiotherapy, it might be a choice to add nimotuzumab to treat NSCLC with LM.

In summary, the findings of this study demonstrate that, within a subset of *EGFR*-mutated NSCLC patients with LM, *EGFR* gene amplification is more likely to occur at the LM stage than at initial diagnosis. Moreover, *EGFR* gene amplification was associated with lower KPS and poorer OS. These findings suggested that *EGFR* gene amplification may be responsible for the resistance of NSCLC–LM patients to *EGFR*-TKIs, and the addition of nimotuzumab will be another choice for the treatment of EGFR-mutated NSCLC patients with LM.

## Data availability statement

The raw data supporting the conclusions of this article will be made available by the authors, without undue reservation.

## Ethics statement

The studies involving human participants were reviewed and approved by the Research Ethics Committee of Guangdong Three Nine Brain Hospital. The patients/participants provided their written informed consent to participate in this study.

## Author contributions

HY, LW, and CS designed the experiments and wrote the manuscript. DL, WH, and XL helped in reviewing, acquiring, analyzing, and interpreting of clinical data for the work. ZZ and ChaZ did the statistical analysis. CheZ, LC, and CaZ critically revised the manuscript for important intellectual content. All authors contributed to the article and approved the submitted version.

## Funding

This work was supported by the National Natural Science Foundation of China (no. 81871865), Natural Science Foundation of Guangdong Province (no. 2019A1515011943), Shanghai Science and Technology Committee Foundation (no. 19411950300), Shanghai Public Health Committee Foundation (NO. 2020CXJQ02) and Medical Scientific Research Foundation of Guangdong Province (no. B2021139). The funders had no role in the study design, data collection and analysis, decision to publish, or preparation of the manuscript.

## Conflict of interest

The authors declare that the research was conducted in the absence of any commercial or financial relationships that could be construed as a potential conflict of interest.

## Publisher’s note

All claims expressed in this article are solely those of the authors and do not necessarily represent those of their affiliated organizations, or those of the publisher, the editors and the reviewers. Any product that may be evaluated in this article, or claim that may be made by its manufacturer, is not guaranteed or endorsed by the publisher.
